# Evaluating the Reporting Quality of Researcher-Developed Alphabet Knowledge Measures: How Transparent and Replicable Is It?

**DOI:** 10.3389/fpsyg.2021.601849

**Published:** 2021-04-16

**Authors:** Sherri L. Horner, Sharon A. Shaffer

**Affiliations:** School of Educational Foundations & Inquiry, Bowling Green State University, Bowling Green, OH, United States

**Keywords:** alphabet knowledge, letter knowledge, research methodology, evaluation, emergent literacy

## Abstract

The American Educational Research Association and American Psychological Association published standards for reporting on research. The transparency of reporting measures and data collection is paramount for interpretability and replicability of research. We analyzed 57 articles that assessed alphabet knowledge (AK) using researcher-developed measures. The quality of reporting on different elements of AK measures and data collection was not related to the journal type nor to the impact factor or rank of the journal but rather seemed to depend on the individual author, reviewers, and journal editor. We propose various topics related to effective reporting of measures and data collection methods that we encourage the early childhood and literacy communities to discuss.

## Introduction

The purpose of educational research is to contribute “to the building, refining, and general acceptance of a core body of knowledge within a collaborative research community” (Lin et al., 2010, p. 299). For this to occur, researchers need to explain their study in ways that enable readers to understand, interpret, and evaluate the quality of the research. “Ernest and McLean (1998) reason that conducting research is akin to detective work and that as researchers we need to provide readers with as many clues as reasonably possible regarding how we have reached our conclusions” (Zientek et al., 2008, p. 208).

Over the past decade or so, the effectiveness of reporting research studies has been scrutinized in various areas of research with many research communities developing standards, policies, or guidelines for their researchers to follow. For instance, in 2010, the medical community revised their Consolidated Standards of Reporting Trials (CONSORT) “to alleviate the problems arising from inadequate reporting of randomized controlled trials” (CONSORT group, 2010, para 1). The European Association of Science Editors (EASE) also developed guidelines in 2010, which they are continuously adapting, to help authors and translators communicate more efficiently in English (European Association of Science Editors, 2018).

Many authors have also investigated different aspects of effective reporting. The journal *Perspectives on Psychological Science* devoted a special issue (see Pashler and Wagenmakers, 2012) to the issues of replicability in psychology. Several researchers investigated the reporting of statistics in journals with different impact factors (Tressoldi et al., 2013) and submission guidelines (Giofrè et al., 2017) while others investigated experimental designs and the statistical methods used (Robinson et al., 2007; Zientek et al., 2008; Zientek and Thompson, 2009; Asendorpf et al., 2013). Still other researchers looked at the publication rates of replication studies (Makel and Plucker, 2014; Makel et al., 2016), or the quality of specific journals (Skidmore et al., 2014; Mohan et al., 2016; Zhao et al., 2017). Several researchers (e.g., Slavin, 2002, 2005; Asendorpf et al., 2013; Makel, 2014; Tressoldi and Giofrè, 2015) have made recommendations to authors, editors, and reviewers related to various elements of published documentation of research. While all of these aspects of reporting are important, the quality of the research rests on the adequacy of the measures and how the data is collected. That is, even if the researcher used appropriate research design, statistical methods, and published in journals with high impact factors, the study is useless if the measures are inappropriate, unreliable, or invalid. We have not found a published article investigating the quality of the measures used or how they are documented. Therefore, we extend the research of documentation of the quality of research by investigating the reporting of researcher-developed measures and data collection methods in published articles specifically relating to alphabet knowledge (AK). Although we are specifically investigating AK measures, our hope is to add to the broader examination of the quality of reporting on research about topics in early childhood and literacy. That is, the quality of reporting on AK measures can serve as a case study for the quality of reporting on measures and data collection methods in general.

In 2006, the American Educational Research Association (AERA) developed standards for reporting on empirical social science research (American Educational Research Association, 2006). The stated purpose for these standards was “to provide guidance about the kinds of information essential to understanding both the nature of the research and the importance of the results” (p. 33). The AERA standards have two overarching principles: (a) warranted and (b) transparent reporting of empirical research. Reporting that uses these principles as guidelines “permits scholars to understand one another's work, prepares that work for public scrutiny, and enables others to use that work” (American Educational Research Association, 2006, p.33). Because we are interested specifically in the transparency of reporting, whether or not the research is warranted is beyond the scope of this article. Two important elements of transparency are interpretability and replicability. That is, is there enough detail in the published article so a reader is able to understand the results and conclusions and duplicate the study?

In 2010, the manual of American Psychological Association (APA) included journal article reporting standards (JARS): information recommended for inclusion in manuscripts that report new data collections regardless of research design (pp. 247–250). The important recommendations from APA JARS related to the topic of this inquiry are (1) definitions of all primary and secondary measures, (2) methods used to collect data, and (3) information on validated or *ad hoc* instruments created for individual studies. Even though (3) of APA JARS relates to both validated and *ad hoc* instruments, we have focused on ad hoc, researcher-developed instruments. We define researcher-developed AK instruments as those that are not published, commercially available measures.

The rationale for focusing on researcher-developed instruments was two-fold. One is that there are different transparency needs for these two types of measures. Authors using validated commercially available measures do not need as many details as those using their own *ad hoc* measures because other researchers can find specific details about these published instruments elsewhere to be able to interpret the results and use the same measure to replicate the study. Also, researcher-developed instruments can lead to an overestimation of the effects of the treatment, which could lead to inflated claims for the results. Therefore, there are more criteria for effective reporting of researcher-developed measures to ensure that the study is interpretable and replicable.

The second rationale is that many researchers of AK rely on their own measures. Approximately 40% of the articles we looked at that had an AK measure used a researcher-developed measure. To our knowledge, there is not, as yet, a validated commercially available assessment of AK only. There are many AK measures as sub-tests of assessments (e.g., Bracken, DIBELS, PALS, OSELA), which can be costly if the researcher needs just the AK subtest. Also, it is easy to develop your own alphabet measure because there is a finite amount of information to assess.

AK is the understanding of various elements of written letters. This includes saying the names of the letters, saying the sounds of the letters, writing the letters, and recognizing the names or sounds of the letters. Each of these types of AK have both uppercase and lowercase letters. Because there are different elements of AK, reporting what exactly was assessed and how it was assessed during a research study is highly important for interpreting the results and conclusions and for replicating the study.

Since Jeanne Chall's research in the 1960's Chall (1967, 1983), AK at entrance to kindergarten has been shown to be highly predictive of future reading ability (Bond and Dykstra, 1967/1997; Share et al., 1984; Adams, 1990; National Research Council, 1998; Lonigan et al., 2000; Storch and Whitehurst, 2002; National Early Literacy Panel, 2008). Therefore, for the past fifty years or so, studies of emergent literacy in pre-school and kindergarten almost always have included an assessment of AK. Many researchers have also used AK as a background or co-variable in studies on literacy development during the elementary school years.

Starting about twenty years ago, literacy researchers have been developing a nuanced understanding of young children's AK. Research (Treiman et al., 1994; Treiman and Broderick, 1998; McBride-Chang, 1999; Lonigan et al., 2000; Evans et al., 2006; Justice et al., 2006; Ellefson et al., 2009; Piasta and Wagner, 2010; Drouin et al., 2012) has shown developmental trends in AK, such as most uppercase letters are typically learned before lowercase, some letters (e.g., O, A) are typically learned before others, and children in the United States (U.S.) typically learn letter names before letter sounds while the reverse is true of British children. Although the picture of alphabet learning is still unclear, there is a growing consensus that not all types of AK are equal in terms of young children's learning. “In practical terms, this means that an assessment of uppercase letter naming, for example, may reveal positively-skewed distributions for children who are 4 years old, normal distributions six months later, and negatively-skewed distributions six months after that” (Drouin et al., 2012, p. 544). For this example, an uppercase letter naming assessment would be useful for only a brief time in a child's development. Plus, AK is even more complicated than this example because it has multiple skills embedded within it (e.g., uppercase naming, lowercase naming, sounds), which have their own developmental trajectories. So, those same U.S. five-year-olds who show a negatively-skewed distribution for uppercase letter naming, may also show a normal distribution for lowercase letter naming, and a positively-skewed distribution for sounds. If researchers only report that they assessed AK, without giving details on what type of AK (e.g., naming, sounds) or type of alphabet letter (i.e., uppercase, lowercase), it is impossible for readers to know exactly what the research entailed. This, in turn, limits how the reader can interpret the data, replicate the study, and apply the results to practice.

Incomplete reporting of research methodology can influence the advancement of the literacy community's knowledge. Ehri et al. (2001), in their meta-analysis of phonics instruction programs, stated “one common weakness of the studies was failing to provide needed information” (p. 431). Piasta and Wagner (2010) did a meta-analysis of alphabet learning and instruction, in which they analyzed the effects of instruction on alphabet outcomes. Their original pool of 300 studies that provided alphabet training and assessed alphabet outcomes was reduced to 37 (12%) studies due to lack of “(1) measurement of discrete alphabet outcomes at post-test, (2) explicit acknowledgment of alphabet training as component of instruction, or (3) sufficient information to calculate an effect size” (p. 10). Therefore, a large number (88%) of the published studies providing alphabet training could not be further analyzed for this meta-analysis because of the non-transparency or lack of interpretability in the reporting. In 2019, Torgerson et al. did a tertiary review of 12 systematic reviews on the effectiveness of phonics interventions. Their review included a quality appraisal using the PRISMA checklist, with five of the 12 articles not reporting doing a quality appraisal of the studies they included. Based on this, Torgerson et al. (2019) stated, “This omission in these five SRs [systematic reviews] is critical, and therefore, the results from these SRs should carry lower weight of evidence in our conclusions (p. 226).” Although Ehri et al. (2001), Piasta and Wagner (2010), and Torgerson et al. (2019) issues related to inclusion were broader than our purposes, these studies highlight the importance of effective reporting in published research articles. A similar lack of transparency related to the reporting of AK measures could restrict the literacy community's ability to accept, build, and refine a core body of knowledge relating to AK development.

A description of the measures in a research study should be “precise and sufficiently complete to enable another researcher, where appropriate, to understand what was done and, where appropriate, to replicate or reproduce the methods of data collection under the same or altered research circumstances” (American Educational Research Association, 2006, p. 35). Therefore, the purpose of this article was to investigate if authors of published articles that use researcher-developed AK measures reported their study sufficiently so it can be interpreted and replicated. Because we are concerned with the interpretability and replicability of research assessing AK, our primary research interest was how authors reported what AK measures they used and how they used them. Our secondary research interest was to examine how authors reported other elements of their study, such as details about their participants and sampling procedures, that are important for interpretability and replicability of the study.

One potential reason for differences in reporting quality is the type of journal in which these articles were published. Perhaps the editors and reviewers for journals that are related specifically to literacy would expect more details and transparency, especially as related to the AK measures, than editors and reviewers for journals that publish on a variety of topics (e.g., early childhood, psychology). Another reason could be that top-tier journals have more rigorous requirements for acceptance and that their reviewers could be more concerned with transparency of reporting than lower-tier journals. Therefore, a tertiary research interest was to examine whether there were differences in the effectiveness of reporting based on type of journal (i.e., literacy and non-literacy) and ranking of journal (e.g., top tier, second tier).

The research questions were as follows:

(1) What percentage of AK elements do authors report in their published articles?
(2) What percentage of General elements do authors report in their published articles?
(3) Are there differences in these percentages based on
  a) type of journal: Literacy journals could be expected to have a higher percentage of AK elements reported while the two journal types would have equal percentages of General elements reported.
  b) ranking of journal: journals with higher impact scores and rankings could be expected to have a higher percentage of AK and General elements reported than journals with lower impact scores and rankings.

## Methods

### Identification of Established Evaluation Criteria

**Figure 1:**
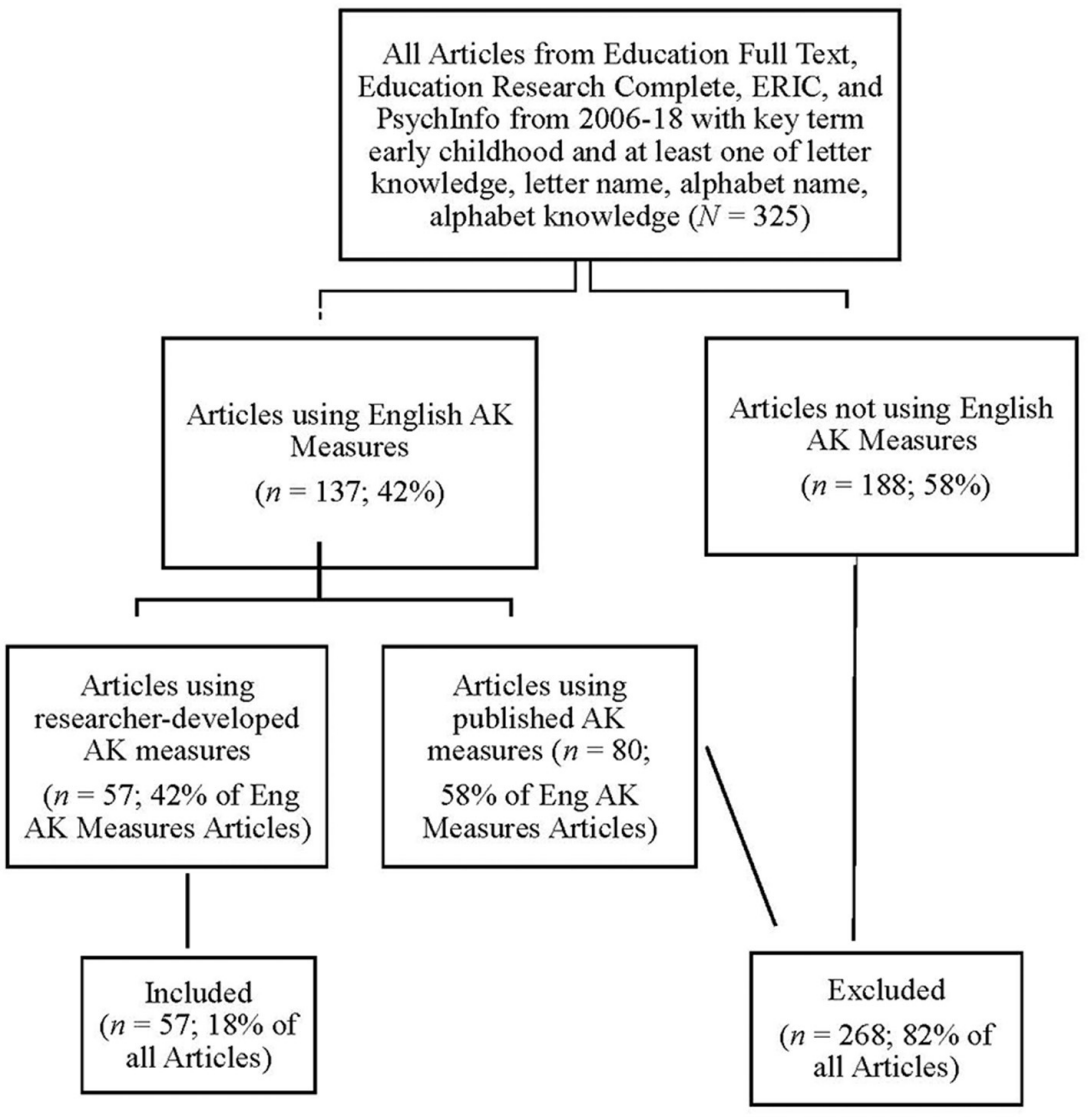
Flow of articles through each stage of inclusionary criteria.

Inclusionary criteria (See Figure 1 for data flow chart) were that the research had to be published in a journal indexed in at least one of the Education Full Text, Education Research Complete, ERIC and PsycINFO databases, with the key term early childhood and at least one of four alphabet-related terms: letter name, letter knowledge, alphabet name, and alphabet knowledge and in the years 2006 to 2018. We chose this time frame because the AERA standards were published in 2006 and 2018 was the last full year of publications when we began this project. Three hundred and twenty-five articles fit these criteria.

Inclusionary criterion for published articles was that the article assessed English AK using researcher-developed measures. This excluded all non-empirical articles (e.g., reviews, advice), plus those that did not include an AK measure (e.g., assessed adults, phonemic awareness), assessed languages other than English (e.g., Dutch, Hebrew) or used published AK measures (e.g., PALS, DIBELS). Fifty-seven articles fit these criteria.

The vast majority of these articles were quantitative (*n* = 51; 89%). The others were single case design (*n* = 3; 5%), case studies (*n* = 2; 4%), and mixed (*n* = 1; 2%) methodologies. Because some elements analyzed were not relevant to all types of methodology, we considered using quantitative methodology as an inclusionary criterion. However, we chose to include all articles using AK measures because many reviews exclude non-quantitative articles and, more importantly, regardless of methodology all articles should be interpretable and replicable.

In reviewing the included articles, we noticed multiple authors had published several journal articles that fit these inclusionary criteria. In a comparison of articles written by the same authors, there were enough differences in the reporting in multiple areas of interest (e.g., types of AK assessed, measures used) to consider these articles as separate entities so all included articles, regardless of authorship, were examined individually.

### Instrumentation

Although we would have preferred to use an existing rubric or checklist (Gall et al., 2007), we were unable to find an existing rubric or checklist that met our purpose for coding the reporting quality of published articles relating to alphabet knowledge measures. Therefore, we developed our own coding rubric based on the American Educational Research Association (2006) standards, American Psychological Association (2010) JARS, and logical deduction of criteria related to the elements of AK. However, to counteract some of the issues with researcher-developed measures, we have attempted to be as transparent as possible in reporting on this measure. The elements of transparency and replicability divided into two categories, which are explained below.

#### AK Measure Reporting

Because of the multi-faceted nature of AK, articles that included the following elements of AK measures would be “precise and sufficiently complete” (American Educational Research Association, 2006, p. 35) for readers to interpret and replicate the study:

1. Definition of AK terms [e.g., alphabet knowledge, letter naming, letter identification; American Psychological Association JARS (2010), Table 1: Measures and covariates: methods used to collect data (p. 842; abbreviated hereafter as APA Table 1: Measures)];
2. Type of knowledge [e.g., names, sounds; American Educational Research Association (2006) standard 3.2 “collection of data or empirical materials” (p. 35; abbreviated hereafter as AERA 3.2)];
3. Type of alphabet letter [i.e., uppercase, lowercase; AERA 3.2];
4. Number of letters used [e.g., all 26, 10; AERA 3.2];
5. Order of letters [e.g., random, letter in participant's name first; AERA 3.2];
6. Format of measure [e.g., one sheet, individual cards; font type and size; AERA 3.2];
7. Directions [e.g., asked child to name letter the examiner pointed to; AERA 3.2];
8. How they used this measure in their analysis [e.g., separate or combined scores; AERA 4.3.c “appropriate use” (p. 36)];
9. methods used to enhance the quality of measurements, including training and reliability of data collectors [AERA 4.2.c. “coding processes”; APA Table 1: Measures];
10. Psychometric properties of researcher-developed instruments [AERA 4.3.c “dependability (reliability)” (p. 36); APA Table 1: Measures].

**Table 1:** Journal information related to AK measures.

**References**	**Read J**	**Quar-tile**	**Impact Factor**	**Type**	**Def**	**type of Know- ledge**	**Type of letter**	**# of letters**	**For- mat**	**Order of letters**	**Direc-tions**	**How Used**	**Train**	**Psy-met**	**Total AK**	**% AK**
Anthony et al. (2009)	Yes	Q1	3.056	Quant	–	Yes	–	Yes	Yes	–	Yes	Yes	Yes	–	6	60%
Arrow and McLachlin (2014)	–	Q2	1.193	Quant	–	Yes	Yes	Yes	Yes	Yes	Yes	Yes	–	Yes	8	80%
Bindman et al. (2014)	–	Q1	2.436	Quant	–	–	Yes	Yes	Yes	Yes	–	Yes	–	Yes	6	60%
Blair and Savage (2006)	Yes	Q1	1.489	Quant	–	Yes	Yes	Yes	Yes	Yes	Yes	Yes	Yes	–	8	80%
Bowyer-Crane et al. (2008)	–	Q1	6.226	Quant	Yes	Yes	–	Yes	–	–	Yes	Yes	Yes	Yes	7	70%
Byrne et al. (2013)	Yes	Q1	3.056	Quant	–	Yes	–	Yes	Yes	–	Yes	Yes	–	Yes	6	60%
Campbell and Mechling (2009)	–	Q1	1.63	SCD	–	Yes	Yes	Yes	Yes	Yes	Yes	Yes	–	–	7	70%
Castles et al. (2013)	–	Q2	1.572	Quant	–	–	–	Yes	Yes	–	–	Yes	–	–	3	30%
Coker and Ritchey (2014)	–	Q2	??	Quant	–	Yes	Yes	Yes	N/a	Yes	Yes	Yes	Yes	Yes	8	89%
Collins and Svensson (2008)	–	Q2	0.81	Qual	–	–	–	–	–	–	–	Yes	–	–	1	10%
Coventry et al. (2011)	–	Q1	1.494	Quant	–	Yes	–	Yes	Yes	Yes	Yes	Yes	–	–	6	60%
Culatta et al. (2007)	–	Q2	0.569	mixed	–	Yes	Yes	Yes	Yes	Yes	Yes	Yes	–	–	7	70%
Cunningham and Carroll (2011)	–	Q1	2.602	Quant	–	Yes	Yes	Yes	Yes	Yes	Yes	Yes	–	Yes	8	80%
Deckner et al. (2006)	–	Q2	2.146	Quant	–	Yes	Yes	Yes	Yes	–	Yes	Yes	–	–	6	60%
Dittman (2016)	Yes	Q1	1.489	Quant	–	Yes	Yes	Yes	Yes	Yes	Yes	Yes	–	Yes	8	80%
Drouin and Harmon (2009)	–	Q1	2.436	Quant	Yes	Yes	Yes	Yes	Yes	Yes	Yes	Yes	Yes	Yes	10	100%
Drouin et al. (2012)	–	Q1	2.436	Quant	Yes	Yes	Yes	Yes	Yes	Yes	Yes	Yes	Yes	–	9	90%
Flynn and Richert (2015)	–	Q2	1.037	Quant	–	Yes	Yes	Yes	Yes	Yes	Yes	Yes	Yes	–	8	80%
Foy and Mann (2013)	Yes	Q1	1.489	Quant	–	Yes	Yes	Yes	Yes	Yes	Yes	Yes	–	–	7	70%
Fuhs et al. (2011)	–	Q2	0.7	Quant	–	Yes	Yes	Yes	Yes	Yes	Yes	Yes	Yes	–	8	80%
Goetz et al. (2008)	Yes	Q1	1.489	Quant	–	Yes	–	Yes	–	Yes	–	Yes	–	–	4	40%
Goldberg and Lederberg (2015)	Yes	Q1	1.489	Quant	Yes	Yes	–	Yes	Yes	Yes	Yes	Yes	Yes	–	8	80%
Greer and Erickson (2018)	Yes	Q1	1.489	Quant	–	Yes	Yes	Yes	Yes	Yes	Yes	N/a	–	–	6	67%
Knell et al. (2007)	–	Q1	1.745	Quant	–	Yes	Yes	Yes	Yes	Yes	Yes	Yes	–	Yes	8	80%
Lerner and Lonigan (2016)	–	Q1	2.146	Quant	–	Yes	Yes	Yes	Yes	Yes	Yes	Yes	Yes	Yes	9	90%
Lonigan et al. (2013)	–	Q1	2.602	Quant	–	Yes	Yes	Yes	Yes	Yes	Yes	Yes	Yes	Yes	9	90%
MacDonald et al. (2013)	–	Q2	3	Quant	Yes	–	–	–	–	–	–	Yes	Yes	Yes	4	40%
McGeown and Medford (2014)	Yes	Q1	1.489	Quant	–	Yes	Yes	Yes	Yes	Yes	Yes	Yes	–	–	7	70%
McGeown et al. (2012)	–	Q1	1.65	Quant	Yes	Yes	Yes	–	–	Yes	Yes	Yes	–	–	6	60%
Neumann (2014)	–	Q1	2.436	Quant	–	Yes	Yes	Yes	Yes	Yes	Yes	Yes	Yes	Yes	9	90%
Neumann et al. (2013a)	Yes	Q1	1.596	Quant	–	Yes	Yes	Yes	Yes	Yes	Yes	Yes	–	–	7	70%
Neumann et al. (2013b)	Yes	Q1	1.489	Quant	–	Yes	Yes	Yes	Yes	Yes	Yes	Yes	Yes	Yes	9	90%
Neumann et al. (2009)	–	Q2	0.74	Case Study	–	Yes	Yes	–	Yes	Yes	Yes	Yes	N/a	N/a	6	75%
Olszewski et al. (2017)	–	Q2	1.321	SCD	Yes	Yes	Yes	Yes	Yes	Yes	–	Yes	Yes	Yes	9	90%
Ouellette and Sénéchal (2008)	Yes	Q1	3.056	Quant	–	Yes	Yes	Yes	Yes	Yes	Yes	Yes	Yes	Yes	9	90%
Phillips et al. (2012)	–	Q1	3	Quant	Yes	Yes	Yes	Yes	Yes	Yes	Yes	Yes	Yes	–	9	90%
Piasta et al. (2016)	–	Q1	1.393	Quant	Yes	Yes	Yes	Yes	Yes	Yes	Yes	N/a	Yes	–	8	89%
Piasta et al. (2010)	Yes	Q1	1.489	Quant	Yes	Yes	Yes	Yes	Yes	Yes	Yes	Yes	Yes	Yes	10	100%
Prior et al. (2011)	–	Q1	1.31	Quant	–	–	–	–	Yes	–	–	–	–	–	1	10%
Rahn et al. (2015)	–	Q2	0.485	SCD	–	Yes	Yes	Yes	Yes	Yes	Yes	Yes	Yes	Yes	9	90%
Ritchey and Speece (2006)	–	Q1	2.877	Quant	–	Yes	Yes	Yes	Yes	Yes	Yes	Yes	–	Yes	8	80%
Roberts et al. (2018)	–	Q1	2.436	Quant	Yes	Yes	Yes	Yes	–	–	–	Yes	Yes	Yes	7	70%
Savage et al. (2007)	–	Q1	6.226	Quant	–	Yes	–	Yes	Yes	–	Yes	Yes	Yes	Yes	7	70%
Schwanenflugel et al. (2010)	Yes	Q1	1.107	Quant	–	Yes	Yes	Yes	Yes	Yes	Yes	Yes	Yes	Yes	9	90%
Shaw and Sundberg (2008)	–	??	??	Quant	–	Yes	Yes	Yes	Yes	–	Yes	Yes	Yes	–	7	70%
Shidler (2009)	–	Q2	0.74	Quant	–	–	–	–	–	–	–	–	Yes	–	1	10%
Smith et al. (2008)	–	Q1	1.25	Quant	–	Yes	–	–	Yes	Yes	Yes	Yes	–	–	5	50%
Strang and Piasta (2016)	Yes	Q1	1.489	Quant	Yes	Yes	Yes	Yes	–	–	Yes	Yes	Yes	Yes	8	80%
Thompson et al. (2015)	–	Q1	3.414	Quant	–	Yes	Yes	Yes	Yes	Yes	–	Yes	–	–	6	60%
Treiman et al. (2013)	Yes	Q1	1.489	Quant	–	Yes	Yes	Yes	Yes	Yes	Yes	Yes	–	–	7	70%
Tyler et al. (2014)	–	Q1	0.55	Quant	–	Yes	Yes	Yes	–	–	–	Yes	Yes	–	5	50%
Wake et al. (2013)	–	Q1	2.393	Quant	–	–	–	Yes	–	–	–	Yes	Yes	–	3	30%
Westerveld (2014)	–	Q1	1.373	Quant	–	Yes	Yes	Yes	Yes	Yes	Yes	Yes	Yes	–	8	80%
Willoughby et al. (2015)	–	Q1	3.819	Quant	–	Yes	Yes	Yes	Yes	Yes	Yes	Yes	–	Yes	8	80%
Wolf (2016)	–	Q2	0.74	Quant	Yes	Yes	Yes	Yes	Yes	Yes	Yes	Yes	–	–	8	80%
Zemlock et al. (2018)	Yes	Q1	1.489	Quant	Yes	Yes	Yes	Yes	Yes	Yes	Yes	Yes	–	Yes	9	90%
Zhang (2018)	–	Q1	1.65	Quant	–	Yes	Yes	Yes	Yes	Yes	Yes	Yes	Yes	Yes	9	90%
Total %					25%	86%	75%	86%	77%	74%	77%	88%	49%	44%		

*AK, Alphabet Knowledge; J, Journal; Q, Quartile; AUS, Australia; CAN, Canada; CHN, China; GBR, Great Britain; HK, Hong Kong; NZ, New Zealand; NOR, Norway; SWE, Sweden; USA, United States; Qual, Qualitative; Quant, Quantitative; SCD, Single Case Design; Def, Definition; Train, Training; Psy-met, Psychometrics*.

#### General Reporting

The elements about the study, which are not specifically related to the measure and data collection methods but need to be disclosed for interpretability and replicability, are:

11. Statement of the purpose or problem under study (AERA 1.1; APA Table 1: Introduction);
12. Research hypotheses or questions (APA Table 1: Introduction);
13. Relevant characteristics of participants: (a) eligibility and exclusion criteria, (b) age/grades, (c) gender (d) race/ethnicity, (e) SES/family income level, (f) language status (e.g., English as a second language), and (g) disability [AERA 3.1.a and APA Table 1: Method: Participant characteristics; although characteristics (b) - (g) are not mentioned specifically, they are relevant because they have been linked to literacy skills].
14. Sampling procedures (a) percentage of sample approached that participated, (b) settings and locations where data were collected, (c) payments made to participants (e.g., money or gifts), and (d) institutional review board agreements and ethical standards met (AERA 3.1.b and APA Table 1: Sampling procedures).

### Coding

Similar to other studies of reporting quality (Zientek et al., 2008; Makel et al., 2016), a dichotomous scale (present, not present) was used and the quality of the information was not evaluated. If the authors mentioned the item anywhere in the article, it was marked as present. If coders could not locate anything related to the item, it was marked as not present except in the following circumstances. Because some elements analyzed were not relevant to all methodologies, a non-applicable (n/a) rather than a not present coding was denoted. Also, some articles investigated one element of AK exclusively so how they used their measure (i.e., individual, combined with other measures) was not applicable. Therefore, percentages, based on only those elements that apply to each study, are reported instead of total scores.

Graduate Assistants (GAs) were trained by the first author. During the training, the coding rubric was explained, then the trainer and trainees coded articles into an excel spreadsheet together until the GAs said they understood the process. The GAs then coded articles independently but were checked by the first author until the coded data were consistent to previously coded information. At least two trained people coded each article. These data sheets were then cross-checked for differences. Of the 1,323 coded items, there were 119 discrepancies (9%; κ = 0.82, an excellent rating; American Psychological Association, 2018). These were then discussed to come to a consensus of whether they were present or not.

## Results

### Reporting Quality

To investigate how authors reported what AK measures they used and how they used them, we looked at the quality of the reporting for the 10 elements of the measures related specifically to AK. See Table 1 for information about the reporting of each element of the AK measure for each article, percentage for each article, and the total percentage for each element. The range was from 10% to 100% of AK elements mentioned. The lowest number of studies (23%) defined the AK terms whereas the highest number of studies (88%, 2 n/a) denoted how they used the AK measure. The average for reporting all 10 AK elements was 71%, with a standard deviation of 22%.

To investigate how authors reported other elements of their study that are important for interpretability and replicability, we looked at the quality of the reporting for the 14 general elements of the studies. See Table 2 for information about the reporting of each general element measure for each article, percentage for each article, and the total percentage for each element. The range was from 36% to 86% of general elements mentioned. The lowest number of studies (18%) mentioned whether any payments were made whereas all authors (100%) stated the purpose for their study and the age of their participants. The average for reporting all 14 general elements was 66%, with a standard deviation of 13%.

**Table 2:** Journal information related to general measures.

**References**	**Country**	**Pur- pose**	**Hy- poth**	**In/Ex clusion**	**Age**	**Sex**	**Ethnic**	**SES**	**ESL**	**Disa-bility**	**% Sam- ple**	**Reg**	**Set**	**Pay**	**Eth**	**Tot Gen**	**% Gen**	**Gr Tot**	**Gr %**
Anthony et al. (2009)	USA	Yes	Yes	Yes	Yes	Yes	Yes	Yes	Yes	Yes	–	Yes	Yes	Yes	–	12	86%	18	75%
Arrow and McLachlin (2014)	NZ	Yes	Yes	Yes	Yes	Yes	–	–	Yes	–	Yes	Yes	–	–	–	8	57%	16	67%
Bindman et al. (2014)	USA	Yes	Yes	Yes	Yes	Yes	Yes	–	Yes	–	–	Yes	Yes	–	–	9	64%	15	63%
Blair and Savage (2006)	CAN	Yes	Yes	Yes	Yes	Yes	–	–	Yes	Yes	Yes	Yes	Yes	–	–	10	71%	18	75%
Bowyer-Crane et al. (2008)	GBR	Yes	Yes	Yes	Yes	Yes	–	Yes	–	Yes	Yes	Yes	–	–	Yes	10	71%	17	71%
Byrne et al. (2013)	AUS/NOR SWE/USA	Yes	–	Yes	Yes	Yes	–	–	–	–	Yes	Yes	Yes	Yes	Yes	9	64%	15	63%
Campbell and Mechling (2009)	USA	Yes	Yes	–	Yes	Yes	–	–	–	Yes	–	–	Yes	–	–	6	43%	13	54%
Castles et al. (2013)	AUS	Yes	–	Yes	Yes	Yes	–	Yes	–	–	Yes	Yes	–	–	–	7	50%	10	42%
Coker and Ritchey (2014)	USA	Yes	Yes	–	Yes	Yes	Yes	Yes	Yes	Yes	–	Yes	Yes	–	–	10	71%	18	78%
Collins and Svensson (2008)	GBR	Yes	Yes	Yes	Yes	Yes	–	Yes	–	–	–	Yes	–	–	–	7	50%	8	33%
Coventry et al. (2011)	AUS/NOR SWE/USA	Yes	Yes	Yes	Yes	Yes	–	–	–	–	Yes	Yes	Yes	–	–	8	57%	14	58%
Culatta et al. (2007)	USA	Yes	–	–	Yes	Yes	Yes	Yes	Yes	Yes	–	Yes	–	–	–	8	57%	15	63%
Cunningham and Carroll (2011)	GBR	Yes	Yes	Yes	Yes	–	–	–	–	–	–	Yes	Yes	–	–	6	43%	14	58%
Deckner et al. (2006)	USA	Yes	Yes	Yes	Yes	Yes	Yes	–	–	–	–	–	Yes	Yes	–	8	57%	14	58%
Dittman (2016)	Aus	Yes	Yes	Yes	Yes	Yes	–	Yes	Yes	Yes	–	Yes	Yes	Yes	Yes	12	86%	20	83%
Drouin and Harmon (2009)	USA	Yes	–	Yes	Yes	Yes	Yes	Yes	Yes	Yes	–	Yes	Yes	Yes	–	11	79%	21	88%
Drouin et al. (2012)	USA	Yes	Yes	Yes	Yes	Yes	Yes	Yes	–	–	Yes	Yes	Yes	–	–	10	71%	19	79%
Flynn and Richert (2015)	USA	Yes	Yes	Yes	Yes	Yes	Yes	Yes	Yes	–	–	Yes	Yes	Yes	–	11	79%	19	79%
Foy and Mann (2013)	USA	Yes	Yes	Yes	Yes	Yes	Yes	–	Yes	Yes	–	–	Yes	–	Yes	10	71%	17	71%
Fuhs et al. (2011)	USA	Yes	Yes	Yes	Yes	Yes	Yes	Yes	Yes	–	–	Yes	Yes	–	–	10	71%	18	75%
Goetz et al. (2008)	GBR	Yes	Yes	–	Yes	Yes	–	–	–	Yes	–	–	–	–	–	5	36%	9	38%
Goldberg and Lederberg (2015)	USA	Yes	Yes	Yes	Yes	Yes	Yes	–	–	Yes	–	–	–	–	–	7	50%	15	63%
Greer and Erickson (2018)	US	Yes	Yes	Yes	Yes	Yes	Yes	–	–	Yes	–	Yes	Yes	–	–	9	64%	15	65%
Knell et al. (2007)	CHN	Yes	Yes	Yes	Yes	Yes	–	Yes	Yes	–	–	Yes	Yes	–	–	9	64%	17	71%
Lerner and Lonigan (2016)	US	Yes	Yes	–	Yes	Yes	Yes	–	–	–	–	Yes	Yes	–	Yes	8	57%	17	71%
Lonigan et al. (2013)	USA	Yes	Yes	Yes	Yes	Yes	Yes	Yes	–	–	–	Yes	Yes	–	–	9	64%	18	75%
MacDonald et al. (2013)	USA	Yes	Yes	Yes	Yes	Yes	Yes	Yes	–	Yes	Yes	Yes	–	Yes	–	11	79%	15	63%
McGeown and Medford (2014)	GBR	Yes	Yes	–	Yes	Yes	–	Yes	Yes	Yes	–	–	–	–	–	7	50%	14	58%
McGeown et al. (2012)	GBR	Yes	Yes	–	Yes	Yes	–	Yes	Yes	–	–	Yes	Yes	–	–	8	57%	14	58%
Neumann (2014)	AUS	Yes	Yes	Yes	Yes	Yes	–	Yes	Yes	Yes	–	Yes	Yes	–	Yes	11	79%	20	83%
Neumann et al. (2013a)	AUS	Yes	Yes	Yes	Yes	Yes	–	Yes	Yes	Yes	–	Yes	Yes	–	Yes	11	79%	18	75%
Neumann et al. (2013b)	AUS	Yes	Yes	Yes	Yes	Yes	–	Yes	Yes	Yes	–	Yes	Yes	–	Yes	11	79%	20	83%
Neumann et al. (2009)	AUS	Yes	N/a	Yes	Yes	Yes	–	Yes	–	Yes	N/a	–	–	N/a	–	6	55%	12	63%
Olszewski et al. (2017)	US	Yes	Yes	Yes	Yes	Yes	Yes	Yes	Yes	Yes	–	Yes	Yes	–	Yes	12	86%	21	88%
Ouellette and Sénéchal (2008)	CAN	Yes	–	Yes	Yes	Yes	–	–	Yes	Yes	Yes	Yes	Yes	–	–	9	64%	18	75%
Phillips et al. (2012)	USA	Yes	Yes	Yes	Yes	Yes	Yes	Yes	Yes	Yes	–	Yes	Yes	–	–	11	79%	20	83%
Piasta et al. (2016)	US	Yes	Yes	Yes	Yes	Yes	Yes	Yes	Yes	Yes	–	Yes	Yes	–	Yes	12	86%	20	87%
Piasta et al. (2010)	USA	Yes	Yes	Yes	Yes	Yes	Yes	–	Yes	Yes	–	Yes	–	–	–	9	64%	19	79%
Prior et al. (2011)	AUS	Yes	Yes	Yes	Yes	Yes	–	Yes	–	Yes	–	Yes	–	–	Yes	9	64%	10	42%
Rahn et al. (2015)	USA	Yes	Yes	Yes	Yes	Yes	Yes	–	Yes	Yes	Yes	Yes	Yes	–	–	11	79%	20	83%
Ritchey and Speece (2006)	USA	Yes	Yes	Yes	Yes	Yes	Yes	Yes	–	–	Yes	Yes	–	–	–	9	64%	17	71%
Roberts et al. (2018)	US	Yes	Yes	Yes	Yes	Yes	–	Yes	Yes	–	–	Yes	–	–	–	8	57%	15	63%
Savage et al. (2007)	GBR	Yes	Yes	Yes	Yes	Yes	Yes	Yes	–	–	Yes	Yes	Yes	Yes	Yes	12	86%	19	79%
Schwanenflugel et al. (2010)	USA	Yes	–	Yes	Yes	Yes	Yes	Yes	Yes	Yes	–	Yes	–	Yes	–	10	71%	19	79%
Shaw and Sundberg (2008)	USA	Yes	Yes	Yes	Yes	–	Yes	Yes	–	–	Yes	–	–	–	–	7	50%	14	58%
Shidler (2009)	USA	Yes	Yes	Yes	Yes	–	Yes	Yes	Yes	Yes	–	Yes	–	Yes	–	10	71%	11	46%
Smith et al. (2008)	USA	Yes	Yes	Yes	Yes	Yes	–	–	Yes	Yes	–	Yes	–	–	–	8	57%	13	54%
Strang and Piasta (2016)	US	Yes	Yes	Yes	Yes	Yes	Yes	Yes	Yes	–	–	–	–	–	Yes	9	64%	17	71%
Thompson et al. (2015)	AUS/NZ	Yes	Yes	Yes	Yes	Yes	–	–	Yes	Yes	–	Yes	–	–	–	8	57%	14	58%
Treiman et al. (2013)	GBR/USA	Yes	Yes	–	Yes	Yes	–	–	Yes	–	–	Yes	–	–	–	6	43%	13	54%
Tyler et al. (2014)	USA	Yes	Yes	Yes	Yes	–	–	–	–	Yes	–	Yes	Yes	–	Yes	8	57%	13	54%
Wake et al. (2013)	AUS/GBR	Yes	Yes	Yes	Yes	Yes	–	Yes	Yes	Yes	Yes	Yes	–	–	Yes	11	79%	14	58%
Westerveld (2014)	NZ	Yes	Yes	Yes	Yes	Yes	Yes	Yes	Yes	–	–	Yes	Yes	–	–	10	71%	18	75%
Willoughby et al. (2015)	CAN	Yes	Yes	Yes	Yes	Yes	Yes	Yes	Yes	–	Yes	Yes	Yes	–	–	11	79%	19	79%
Wolf (2016)	US	Yes	Yes	Yes	Yes	Yes	–	Yes	–	–	–	–	Yes	–	Yes	8	57%	16	67%
Zemlock et al. (2018)	US	Yes	Yes	Yes	Yes	Yes	–	Yes	–	–	–	Yes	Yes	–	Yes	9	64%	18	75%
Zhang (2018)	Hong Kong	Yes	Yes	Yes	Yes	Yes	Yes	Yes	Yes	–	–	Yes	Yes	Yes	Yes	12	86%	21	88%
Total %		98%	86%	84%	98%	91%	50%	64%	59%	53%	26%	81%	60%	19%	31%				

*Tot, Total; AK, Alphabet knowledge; Gen, General; Hypoth, Hypotheses; SES, Socio-economic status; Sam, Sample; Sel, Selection; Reg, Region; Set, Setting; Pay, Payment; Eth, Ethics; Gr, Grand*.

To investigate how authors reported all elements of their study, we looked at the overall quality by combining the 24 elements. The total percentage ranged from a low of 33% to a high of 88%, with an average of 68% and standard deviation of 13%.

### Type of Journal

To investigate whether there were differences in the level of reporting based on type of journal, we divided the articles into literacy and non-literacy publications. There were four literacy journals: *Journal of Literacy Research* (*n* = 1 article), *Reading and Writing: An Interdisciplinary Journal* (*n* = 12), *Reading Research Quarterly* (*n* = 1), and *Scientific Studies of Reading* (*n* = 3), with 17 articles total. There were 30 non-literacy journals, with 40 articles total. The *Early Childhood Research Quarterly* had five articles, *Early Childhood Education Journal* and *Journal of Experimental Child Psychology* had three, *Journal of Child Psychology and Psychiatry* and *Learning and Individual Differences* had two, and 25 journals published only one article each from 2006–2018.

Independent t-tests were run for AK, General, and total percentages with a 95% confidence interval (CI) for the mean difference. We used Hedges' *g* to calculate the effect size because the sample sizes were different. As can be seen in Table 3, the literacy journals' average for AK measures (*M* = 76%) was higher than non-literacy journals' (*M* = 69%); however, this difference was not statistically significant, *t*_(55)_ = 1.15, *p* = 0.257, Hedges' *g* =.033. For the General elements, the literacy and non-literacy journals' averages were almost the same (*M* = 65, 66%, respectively) so there was no statistical significance, *t*_(55)_ = −0.24, *p* = 0.813, Hedges' *g* = 0.069. As to be expected from the previous results, the total averages (literacy *M* = 70%; non-literacy *M* = 67%) were also not statistically significant, *t*_(55)_ =.64, *p* = 0.524, Hedges' *g* = 0.186. The results for the General elements agreed with our prediction that the differences would not be statistically significant. Our prediction that the literacy journals would be more precise in their reporting of AK elements was not supported. However, because of the small number of articles analyzed the statistical power for these analyses was not high.

**Table 3:** Averages and standard deviations for elements of reporting by type and quality of journal.

	**Articles**		**AK**		**General**		**Total**	
**Journal type**	**Number**	**Percentage**	**M**	**SD**	**Mean**	**Std. Dev**	**Mean**	**Std. dev**
Literacy	17	29.82%	75.69%	14.71%	65.13%	14.03%	69.52%	11.79%
Non-literacy	40	70.18%	68.57%	23.67%	66.01%	12.24%	67.06%	13.78%
Total	57		70.69%	21.52%	65.74%	12.68%	67.80%	13.17%
Quartile								
1st	42	75.00%	73.23%	18.56%	66.16%	12.92%	69.10%	12.20%
2nd	14	25.00%	63.13%	28.72%	65.63%	12.11%	64.57%	15.92%
Total	56		70.70%	21.71%	66.03%	12.61%	67.97%	13.22%

*AK, Alphabet Knowledge*.

### Journal Impact Factors and Ranking

To investigate whether there were differences in the level of reporting based on type of journal, we divided the articles by two common ways to evaluate journals; thereby, allowing two different statistical analyses. We used impact factor scores as reported on the individual journals' websites or researchgate.com. We could not locate impact factors for two journals (*Assessment for Effective Interventions* and *Journal of At-Risk Issues*) so 55 articles were in this analysis. Because impact factor scores are continuous, we ran correlations between this measure and the two elements of reporting. Impact factor was not significantly correlated with AK, General, or Total scores.

We also used Scimago Journal Rank (SCImago, 2020), which “is a publicly available portal that includes the journals and country scientific indicators developed from the information contained in the Scopus® database (Elsevier B.V.).… Citation data is drawn from over 21,500 titles from more than 5,000 international publishers” (no page). We could not locate a quartile ranking for one journal, *Assessment for Effective Interventions*, so 56 articles were in this analysis. Forty-two (75%) journals were ranked in Quartile 1 (Q1) while 14 (25%) were in Quartile 2 (Q2).

Independent t-tests were run for AK, General, and total percentages with a 95% confidence interval (CI) for the mean difference. We used Hedges' *g* to calculate the effect size because the sample sizes were different. As can be seen in Table 3, the Q1 journals' averages for all measures (AK *M* = 73%, General *M* = 66%; Total *M* = 69%) were higher than Q2 journals' (AK *M* = 63%, General *M* = 66%; Total *M* = 65%); however, these differences were not statistically significant, *t*_(54)_ = 1.52, *p* = 0.133, Hedges' *g* = 0.470; *t*_(54)_ = 0.13, *p* = 0.894, Hedges' *g* =.042; *t*_(54)_ = 1.11, *p* = 0.270, Hedges' *g* = 0.343 respectively. Therefore, our prediction that higher-tier journals would be more precise in their reporting than lower-tier journals was not supported. However, because of the small number of articles analyzed the statistical power for these analyses was not high.

Although interactions between type (i.e., literacy and non-literacy) and journal ranking (i.e., Q1 and Q2) could be another potential reason for differences in the level of reporting, we could not investigate this. All four literacy journals were ranked in the first quartile; therefore, we were unable to differentiate between ranking and type of journal.

### Summary

Over the past decade, multiple organizations, including AERA and APA, have developed or revised standards for testing, evaluating, and reporting research. Although there has been increased awareness of these issues by organizations, the degree to which journal articles aligned with these standards fluctuated greatly, with a low of 33% of elements mentioned to a high of 88%, with an average of 68%. The contrast was even greater for just the AK measures with a range from 10 to 100%, with an average of 71%. There was no statistically significant relationship between the quality of reporting and the type (i.e., literacy or non-literacy) or evaluation (i.e., impact factor, ranking) of the journal.

## Discussion

Effective reporting is an important but overlooked element of research dissemination. Even if the research itself is rigorous, if the authors omit crucial details in the reporting of their study, the published article is flawed, with decreased interpretability and replicability. This, in turn, can lead to problems with the literacy research community's ability to accept, build, and refine (Lin et al., 2010) a core body of knowledge relating to AK development.

In this study, we have shown that the quality of reporting details of the AK measure and general information as recommended by AERA and APA JARS standards differ from published article to article. These differences are not due to being published in a literacy-oriented journal instead of other journals nor top-tier rather than second-tier journals. Our finding is similar to Tressoldi et al. (2013) who found that “statistical practices vary extremely widely from journal to journal, whether IF [impact factor] is high or relatively low” (p. 6).

There are several possible reasons for these differences: authors themselves, the reviewers of the manuscripts, the editors of the journals, or a combination of the three. The majority of articles (*n* = 40; 70%) were authored by researchers who published only once from 2006–2018. There were five sets of authors with more than one publication, with all except for Drouin publishing in multiple journals. Co-authors Anthony, Lonigan, and Piasta, in various combinations of authorship, published seven articles with a range of 71-87%. Neumann (*n* = 4) ranged from 63-83%. McGeown's two articles were both 58%, Drouin's were 79 and 88%, and Bryne's were 58 and 63%. Therefore, there is some fluctuation in the level of effective reporting even within the same authorship, although it is not as wide as for the whole sample (33–88%). Only McGeown's two articles had the exact same level of effectiveness; therefore, at least some of the reason for this fluctuation lays with the authors themselves.

The majority of journals (*n* = 27; 82%) published only one article with researcher-developed AK measures from 2006 to 2018. Six journals had more than one publication. *Reading and Writing: an Interdisciplinary Journal* published 12 articles, with a range of 38-83%. *Early Childhood Research Quarterly* (*n* = 5) ranged from 63 to 88%, *Scientific Studies of Reading* (*n* = 3) were between 63 and 75%, *Early Childhood Education Journal* (*n* = 3) were 46-67%, and *Journal of Experimental Child Psychology* (*n* = 3) ranged from 58 to 75%. *Journal of Child Psychology and* Psychiatry's two articles were 71% and 79% while *Learning and Individual Differences'* were 71 and 75%. Similar to authorship, there is some fluctuation in the level of effective reporting even within the same journal, although it is not as wide as for the whole sample (33–88%). However, *Reading and Writing*, the journal with the most publications, had the widest range and was most similar to the whole sample. Therefore, at least some of the reason for this fluctuation lays with the journal. Although we have no way of knowing what the authors, reviewers, and editors' interactions were, it seems like the fluctuations in reporting detail can be best accounted for by a combination of all three.

With the development of the standards, AERA and APA organizations have “provided guidance about the kinds of information essential to understanding both the nature of the research and the importance of the results” (American Educational Research Association, 2006, p. 33). However, even after 8–12 years, as can be seen from our analysis, many articles lack key elements of these standards. Perhaps these researchers are not aware of or use these standards. Or, because these standards are recommendations not imperatives, researchers might use some but not all of them. Therefore, the effectiveness of reporting still depends on individual authors, reviewers, and editors. The total overall level of effective reporting on early childhood measures, including AK, and general information could be enhanced if the early childhood research community developed some guidelines specific to research with young participants. In the following paragraphs, we suggest some questions the research community could use to discuss ways in which we could develop specific guidelines to enhance the AERA and APA standards.

One question for this potential discussion would be whether all elements within the AERA and APA standards should be treated as equally important or whether there some elements that are more relevant to early childhood than others. In this study, we treated all elements as equally important, with each element worth one point. But, are all these elements equally necessary for transparence or are some more vital? For instance, prior research has shown that young children typically learn uppercase before lowercase, U.S. children learn names before sounds while British learn sounds first, and some letters are typically learned before others (Treiman et al., 1994; Treiman and Broderick, 1998; McBride-Chang, 1999; Lonigan et al., 2000; Evans et al., 2006; Justice et al., 2006; Ellefson et al., 2009; Piasta and Wagner, 2010; Drouin et al., 2012); therefore, we think letter type, knowledge type, number of letters and how they are used all are vital for interpretation. The other aspects (e.g., format, directions) could enhance replicability but probably are not necessary for understanding the results. However, this is our opinion so we urge the research community to discuss what elements are necessary to report for transparency.

What is necessary to know about participants? All studies (100%) reported age, 53 (93%) reported sex, and 49 (86%) mentioned who was included or excluded from the study. Ethnicity, SES, English status, and whether anyone had a disability were mentioned by 51-65%. Are all these elements necessary to report in all studies or are they only necessary if they are an important aspect of the study? For instance, English status and disabilities were, of course, mentioned when they were an integral part of the research, such as the participants were children with Down's syndrome or English Learners (EL). They were also mentioned frequently as exclusionary criteria (e.g., children with speech impediments were not tested). However, if studies did not mention these elements does it reduce the readers' ability to interpret and replicate the study substantially? If these aspects are not mentioned, do readers assume the researchers did not record this information or there were no participants with disabilities or EL? Do these assumptions change readers' understanding or interpretation of the results?

Reporting of the ethnicity of participants brings up a unique and interesting issue for discussion. It was included in our study because previous research has linked it to reading achievement. We found that whether authors reported it or not depended, at least in part, on where they did their study. Of the 30 studies done exclusively in the United States, 81% mentioned ethnicity whereas only four of the 23 (17%) studies done in other countries and zero of the three studies that were done in the United States and other countries did so. No other element had this division by country. This adds a potential twist to the discussion for guidelines – could the criteria for effective reporting depend on various non-research factors, like country?

Another aspect, beyond what elements should be included in articles, of this potential discussion is the quality of the reporting. We did not assess the quality of the reporting; that is, any mention of an element was given a point. However, is there a level of detail that is ideal? For instance, should readers assume some basic level of ethics is met if the article is published or does it have to always be explicitly stated? Especially when considering page limitations of most journals, is it necessary for authors to include, for example, information about IRB approval or other ethical treatment of participants in all journal articles? Or, for research that does not include elements that could be harmful to participants, such as asking about alphabet letters, can the ethics of the research be monitored by the editor and assumed by the readers? Authors could mention any ethical considerations in a cover letter but save space in the manuscript.

Another area of concern is training. Some authors gave detailed explanations of the training whereas others included the word “trained.” Should readers trust that the assessors were adequately trained or should we require more details? Should these requirements differ by measure? That is, training assessors for an alphabet knowledge measure would be easier than for analyzing poetry writing. Even within alphabet knowledge, letter naming is straight-forward with English-speaking adults knowing the names (except perhaps the American zee and the Queen's English zed) and how to score them whereas letter sounds is more complex, with multiple sounds for some letters including long and short vowels. Again considering page limitations, how detailed does the description of training need to be?

Another major question, if there is a discussion with guidelines developed, is how does the research community disseminate this information? The articles using researcher-developed AK measures are widely dispersed. From 2006 to 2018, there were 57 articles published in 34 journals, ranging from medical, communication disorders, early childhood, psychology, and literacy. Because of this variety, reviewers might not be knowledgeable about AK and the specific requirements for interpretability and replicability. Therefore, how to make sure authors, reviewers, and even editors are knowledgeable about the necessary components to describe becomes complicated and important.

These are just some of the areas that the early childhood research community could discuss and use to help formulate guidelines for publication specific to our particular genre. Although we looked specifically at AK with many of the details investigated distinctly related to AK (e.g., type of letter), we believe other literacy-related (e.g., phonemic awareness, vocabulary, fluency) and early childhood (e.g., social skills, executive functioning) knowledge and skills would also need details on researcher-developed measures to be transparent, interpretable, and replicable. Although we cannot determine without doing a similar study for other bodies of research, we would not be surprised if other research areas had the similar levels of fluctuation in the transparency of the reporting of their researcher-developed measures and data collection methods. Therefore, we encourage the literacy and early childhood research communities to engage in a rigorous discussion of these issues surrounding effective reporting of research. In this way, we can add our specific guidelines to the broader standards to “to assist researchers in the preparation of manuscripts that report such work, editors and reviewers in the consideration of these manuscripts for publication, and readers in learning from and building upon such publications” (American Educational Research Association, 2006, p. 33).

## Data Availability Statement

The original contributions presented in the study are included in the article/supplementary material, further inquiries can be directed to the corresponding author.

## Author Contributions

SH coded and input data, analyzed the data, did the statistical analysis, wrote some of the manuscript, proofread/edited the manuscript, submitted. SS did the literature searches, collected the articles, coded and input data, analyzed the data, wrote some of the manuscript, proofread/edited the manuscript. All authors contributed to the article and approved the submitted version.

## Conflict of Interest

The authors declare that the research was conducted in the absence of any commercial or financial relationships that could be construed as a potential conflict of interest.
